# Next-generation sequencing facilitates differentiating between multiple primary lung cancer and intrapulmonary metastasis: a case series

**DOI:** 10.1186/s13000-021-01083-6

**Published:** 2021-03-11

**Authors:** Changjiang Liu, Chengang Liu, Xiao Zou, Lin Shao, Ying Sun, Yang Guo

**Affiliations:** 1 Department of Thoracic Surgery, Fourth Hospital of Hebei Medical University, Shijiazhuang, 050000 China; 2 Burning Rock Biotech, Guangzhou, 510300 China

**Keywords:** Multiple pulmonary nodules, Multiple primary lung cancer, Intrapulmonary metastasis, Next-generation sequencing; case report

## Abstract

**Background:**

In lung cancer management, differential diagnosis between multiple primary lung cancer (MPLC) and intrapulmonary metastasis (IMP) is a critical point that is of direct therapeutic and clinical importance. However, this process often suffers from absence of a gold standard, resulting in equivocal cases. Herein, we present a series of three cases, in which genomic alteration patterns revealed by next-generation sequencing (NGS) facilitated the differential diagnosis between MPLC and IMP.

**Case presentation:**

Case 1 was a 57-year-old female with two separate lesions in the upper lobe and the lower lobe of left lung, which were both histopathologically determined as T2aN0M0 adenocarcinomas. NGS identified an *EGFR* L858R in one lesion and an *EGFR* 20 exon insertion in the other one, suggestive of double primary malignancies. The patient underwent wedge resections and received an adjuvant treatment of icotinib and chemotherapy. She had a disease-free survival (DFS) of 19 months and counting. Case 2 was a 55-year-old female with multiple small lesions in both lungs. Histopathological examinations of resected lesions from right upper lobe revealed three subtypes: atypical adenomatous hyperplasia of alveolar epithelium, adenocarcinomas in situ and minimally invasive adenocarcinoma. NGS identified two different *BRAF* driver mutations G466E and V600_K601delinsE in two lesions of adenocarcinoma in situ, and a *BRAF* K601E in a lesion of minimally invasive adenocarcinoma. Case 3, a 68-year-old male, had the right upper lobe lesion histophathologically classified as a stage T3NxM0 mixed adenoneuroendocrine carcinoma and the left upper lobe lesion as a stage T1aN0M0 adenocarcinoma. NGS performed with different loci of surgical tissues revealed a rare sensitizing *EGFR* mutation G719A shared by the right upper lobe lesion and lymph node, and two *EGFR* mutations L861Q and G719S in left upper lobe lesion. The patient received icotinib treatment postoperatively and achieved a stable disease with a progression-free survival of 5 months.

**Conclusion:**

Our cases provide evidence for utility of NGS in facilitating diagnosis and treatment decisions.

**Supplementary Information:**

The online version contains supplementary material available at 10.1186/s13000-021-01083-6.

## Background

Multiple lung cancers, manifested mainly as anatomically separated pulmonary nodules in imaging findings, have been a long-standing issue confronting pathologists and thoracic surgeons, as accurate staging of these nodules would have both therapeutic and prognostic significance [1]. If the multiple lesions are diagnosed as multiple primary lung cancers (MPLC), the patient would mainly undergo curative resection. By contrast, if intrapulmonary metastasis (IPM) is found present, the patient would be more likely to benefit from systemic treatment modalities such as chemotherapy or targeted therapy [2]. Reported incidence of multiple lung nodules ranges from 5 to 20% in studies of varying samples sizes and populations [3, 4]. Moreover, with the widespread launch of large-scale cancer screening projects equipped with high-resolution imaging devices, multiple lung nodules are becoming increasingly detected. Therefore, there is a pressing need for a diagnostic workup that effectively distinguishes MPLC from IPM.

However, current tumor staging system, which largely rely on histological and pathological features, lacks the standard criteria for the diagnosis of MPLC. This situation at times leads to equivocal cases, in which the lung nodules are histologically and pathologically the same or highly similar [3, 5]. To facilitate diagnosis of these difficult cases, a number of molecular subtyping tools have been employed, including comparative genomic hybridization (CGH), loss of heterogeneity analysis (LOH), and detection of mutations in hotspot genes based on polymerase chain reaction or next generation sequencing (NGS) [5–7]. In particular, advances in NGS technology have enabled a more sensitive and economic analysis with higher throughput. As a result, NGS has attracted growing attention as a useful adjunct to the histopathological diagnostic workup, especially in lung cancer, for which molecular subtyping routinely assists therapeutic choices.

Here, we report a series of three Chinese patients for whom targeted NGS profiling provided key additional knowledge that allowed for definitive diagnosis of MPLC. All three patients had their resected pulmonary lesions genomic profiled with a panel of 520 cancer-related genes. The distinctive genomic profiles in different pulmonary lesions lend strong support to definitive diagnosis of MPLC rather than IPM.

We present the following cases in accordance with the CARE reporting checklist.

## Case presentation

### Case 1

**Fig. 1:**
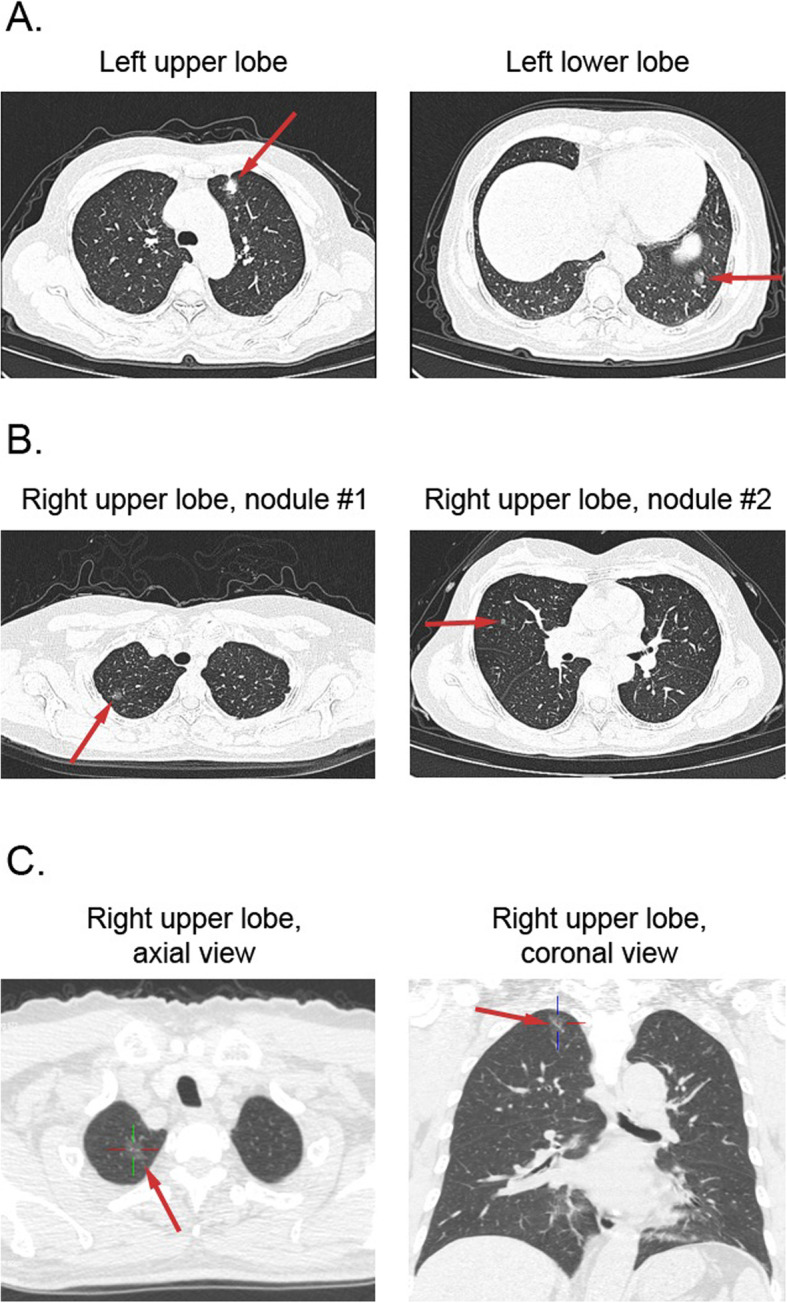
Computed tomography (CT) scans showing pulmonary nodules detected in the cases presented in this series. **a**. In Case 1, two nodules of similar sizes (arrows) were observed in the two lobes of the left lung; **b**. A large number of small nodules were found in both lungs in Case 2. Shown here are two of the largest lesions; **c**. Transverse and frontal views of a nodule in the right upper lobe in Case 3. More miliary micronodules, although not readily discernable in CT scans, were discovered during surgical removal of this nodule

A 57-year-old female patient with continuous coughing for 1 month underwent a chest CT scan, which revealed two nodules in the left lung: one in the upper lobe (1.8 cm in diameter,) and one in the lower lobe (1.5 cm in diameter) (Fig. 1a). In Jan, 2019, the patient underwent wedge resection of the upper and lower lobes of left lung under thoracoscope. Pathological examinations determined a stage T2aN0M0 adenocarcinoma for both lesions. NGS profiling of two resected lesions was performed using a panel of 520 cancer-related genes (OncoScreen, Burning Rock Biotech, Guangzhou, China) on a NextSeq 500 (Illumina, Inc., San Diego, CA, USA). The 520 genes were listed in Additional file 1. The materials and method of NGS were also described in Additional file 2. The results showed that one lesion harbored an *EGFR* sensitizing mutation L858R accompanying with mutations in *TP53*, *CTNNB1*, *PDGFRB* and *SMARCA4*, as well amplifications in *SDHA* and *TERT*; the other one had an *EGFR* 20 exon insertion and concomitant mutations in *ARID1A* and *ASXL2* (Table 1). The distinctive genomic patterns in two lesions supported the double primary malignancies. The patient was treated with an adjuvant therapy of icotinib and chemotherapy and has currently achieved a disease-free survival (DFS) of 19 months.

**Table 1:** Genomic alterations detected by NGS profiling of pulmonary resections from three cases

Case #	1		2			3			
**Specimen #**	1	2	1	2	3	1	2	3	4
**Specimen source**	LUL	LLL	lesion #2	lesion #5	lesion #6	RUL	RUL	LN	LUL
**Tumor cell fraction**	80%	40%	8%	10%	20%	80%	10%	40%	70%
**Mutation**									
*EGFR*									
L858R	+								
20in S768_D770dup		+							
E709K						+	+	+	
G719A						+		+	
G719S									+
L861Q									+
*BRAF*									
G466E			+						
V600_K601delinsE				+					
BRAF K601E					+				
*TP53*									
P278A	+								
R248W									+
R273L						+	+	+	
*CHEK2*									+
*CTNNB1*	+								
*ARID1A*		+							
*ASXL2*		+							
*FLT4*			+						
*HOXB13*						+		+	
*KMT2D*				+					
*MAP 2 K4*							+	+	
*NF1*			+						
*PALB2*						+		+	
*PAX5*						+	+	+	
*PDGFRB*	+								
*RB1*						+			
*SMARCA4*	+								
*TET2*					+				
**Amplification**									
*CCND3*									+
*CDK4*						+		+	
*CDKN1A*									+
*DAXX*									+
*FANCE*									+
*GLI1*						+		+	
*GNAS*						+			
*MDM2*						+		+	
*NKX2–1*						+		+	
*NOTCH4*									+
*PAK1*						+	+	+	
*PIM1*									+
*SDHA*	+								
*TERT*	+					+			
*VEGFA*									+

*NGS* Next-generation sequencing, *LUL* Left upper lobe, *LLL* Left lower lobe, *RUL* Right upper lobe, *LN* Lymph nodes. +, tested positive for indicated alteration

### Case 2

A 55-year-old female, following the detection of multiple small nodules in both lungs by CT scan (Fig. 1b), underwent wedge resection of left and right upper lobes in Jan. 2019. The surgery removed a total of six big lesions in the right upper lobe. Lesions 1 and 4 were pathologically diagnosed as atypical adenomatous hyperplasia of alveolar epithelium, lesions 2, 3, and 5 as lung adenocarcinomas in situ (stage TisN0M0), and lesion 6 as minimally invasive lung adenocarcinoma (T1miN0M0). NGS profiling with the same 520-gene panel was performed on six lesions, three of which (nos. 1, 3, and 4) had no genomic alteration detected probably due to the low tumor cell fraction in the samples. Lesions 2 and 5, which had the same histology of adenocarcinomas in situ, harbored the *BRAF* driver mutation G466E and V600_K601delinsE, respectively, while lesion 6 harbored a *BRAF* K601E (Table 1, Additional file 3). Of note, this is the first report of multiple primary cancer patient with different *BRAF* driver mutations. Different accompanying mutations were also identified in the three lesions.

### Case 3

A 68-year-old male patient was detected with a small pulmonary nodule in the right upper lobe (2 cm in diameter, Fig. 1c). He underwent resection with curative intent under thoracoscope in November, 2018. However, miliary micronodules on the surfaces of both lungs were discovered during surgery, along with multiple enlarged lymph nodes on superior mediastinal. Wedge resection of the left and right upper lobes was therefore performed, removing the two largest lesions. The pathogenic examinations of the surgical tissues identified the right upper lobe lesion (2 cm in diameter) as a stage T3N1M0 mixed adenoneuroendocrine carcinoma, while the left upper lobe lesion (1 cm in diameter) was classified as a stage T1aN0M0 adenocarcinoma. NGS using the 520-gene panel was performed for 4 specimens from different loci of surgical tissues including 2 from the right upper lobe (no.1 and 2), 1 from paratracheal lymph node (no.3) and 1 from left upper lobe (no.4). Specimens no.1, 2, and 3 revealed a highly similar genomic signature sharing nearly identical alterations, including a rare sensitizing *EGFR* mutation G719A (Table 1); while specimen no.4 harbored totally different genomic alterations, including two rare sensitizing *EGFR* mutations L861Q and G719S, suggestive of a different origin. The patient started icotinib treatment since December, 2018, and achieved a stable disease with a progression-free survival of 5 months. After progressing on icotinib, the patient received an additional combined chemotherapy of etoposide and lobaplatin from June, 2019 to January, 2020. He developed a progressive disease in March, 2020 and passed away in June 2020 with an overall survival of 19 months.

## Discussion and conclusions

While staging pulmonary lesions is generally consistent among major tumor grading guidelines, a considerable degree of uncertainty has long been noted in differentiating between MPLC and IMP [8]. In cases without clear-cut histopathological distinctions, concordance of genomic alteration profiles between multiple nodules provides additional insight into their clonal relationship, and therefore can often lead to a definitive diagnosis. In our study, Case 1 had two lesions with similar sizes (1.8 cm and 1.5 cm in diameter, respectively), both being adenocarcinomas and invading the visceral pleura, which would force a close call without further evidence. However, NGS reveals two entirely different profiles. Of note, a *EGFR* driver mutation L858R was detected only in one lesion, which has been reported to rarely exhibit discordant patterns between primary tumor and associated metastasis [9]. Findings from targeted NGS has therefore facilitated a definite diagnosis of MPLC. Similarly in case 2, NGS also identified different *BRAF* driver mutations and distinct accompanying alteration profiles in histologically-identical lesions in the same lobe, which likely would have be misdiagnosed as IMPs.

In addition to diagnosis of multiple primary tumors, mutation profiles also provide hints for the origin of lymph node metastasis. In case 3, NGS discovered divergent rare *EGFR* mutations in the right upper lobe lesion versus in the left upper lobe lesion, while the mutations profile of the upper paratracheal lymph nodes was highly similar to that of the former, suggesting that the lymph node metastasis was originated from the right upper lobe lesion and further confirming the staging of the tumor (T3N1M0).

Compared with other molecular approaches such as CGH and LOH, NGS offers unique advantages. The underlying rationale of applying mutational analysis in distinguishing MPLC from IMP is the postulation that concordance in mutation status is a surrogate of clonal relationship. In practice, however, identification of genomic alterations is typically confounded by the combination of small sample amount, low tumor cell fraction, and “passenger mutations”. These limitations hold especially true for cases where surgical biopsy yields small sample amounts or is deemed not feasible. In this scenario, the multiplexed nature of NGS technology, which leads to high throughput and sensitivity, makes it a competent addition to the current diagnostic workup. Indeed, a recent study of 120 patients demonstrated that molecular subtyping could lead to more sensitive detection of MPLA than with histopathological features, and proposed the use of a combined histomolecular algorithm for differentiating between MPLC and IMP [10]. Besides, NGS also provides additional information for therapeutic decisions. Case 1 and 3 both received icotinib treatment on the basis of the detection of *EGFR* sensitizing mutations, and both achieved favorable clinical outcomes.

In conclusion, we present a series of three cases, where diagnoses of MPLC were made with the help of markedly different genomic alteration profiles. Large prospective studies are warranted to further establish a more powerful diagnostic workup that incorporates radiologic, histopathological, and molecular examinations in distinguishing MPLC from IMP.

## Supplementary Information

**Additional file 1.** List of the 520 genes in the OncoScreen panel.

**Additional file 2.** Supplemental Materials and methods for DNA isolation and capture-based targeted DNA sequencing.

**Additional file 3.**IGV pictures illustrating *BRAF* p.K601E and V600_K601delinsE mutations.

## Data Availability

The datasets used and/or analysed during the current study are available from the corresponding author on reasonable request.

## Abbreviations

MPLC: Multiple primary lung cancer
IMP: Intrapulmonary metastasis
NGS: Next-generation sequencing
CGH: Comparative genomic hybridization
LOH: Loss of heterogeneity
