# Supplementary material for: Next-generation sequencing facilitates differentiating between multiple primary lung cancer and intrapulmonary metastasis: a case series
Source: Diagn Pathol. 2021 Mar 11;16:21. doi: 10.1186/s13000-021-01083-6 (PMC7953741; doi:10.1186/s13000-021-01083-6)
Supplement: Supplementary file 2 — Additional file 2. Supplemental Materials and methods for DNA isolation and capture-based targeted DNA sequencing. [file 13000_2021_1083_MOESM2_ESM.docx]

**Supplemental materials and methods**

**DNA isolation and capture-based targeted DNA sequencing**

Genomic DNA was extracted from fresh surgical tissues or formalin-ﬁxed, parafﬁn-embedded (FFPE) tumor tissues using a QIAamp DNA FFPE tissue kit (Qiagen, Hilden, Germany). according to the manufacturer’s standard protocol. A minimum of 50mg fresh surgical tissues or 5 slides of FFPE samples was required. A minimum of 50 ng of DNA was used for NGS library preparation. Isolated DNA was sheared using Covaris M220 (Covaris, MA, USA) and then subjected to end repair, phosphorylation, and adapter ligation. Fragments between 200–400 bp in size from the sheared genomic DNA were selected, puriﬁed using beads (Beckman Coulter, CA, USA) and hybridized with capture probes of a panel consisting of 520 cancer-related genes spanning 1.64 megabases (Mb) of the human genome (Burning Rock Biotech, Guangzhou, China). The quality and size of the library were assessed using a Qubit 2.0 ﬂuorometer with the dsDNA high-sensitivity assay kit (Life Technologies, Carlsbad, CA). Indexed samples were sequenced on Nextseq500 (Illumina, Inc., USA) with paired-end reads and a median sequencing depth of 1000×.
