# Supplementary figures and images for: Next-generation sequencing facilitates differentiating between multiple primary lung cancer and intrapulmonary metastasis: a case series
Source: Diagn Pathol. 2021 Mar 11;16:21. doi: 10.1186/s13000-021-01083-6 (PMC7953741; doi:10.1186/s13000-021-01083-6)

**Illustration of BRAF p.K601E and V600_K601delinsE**


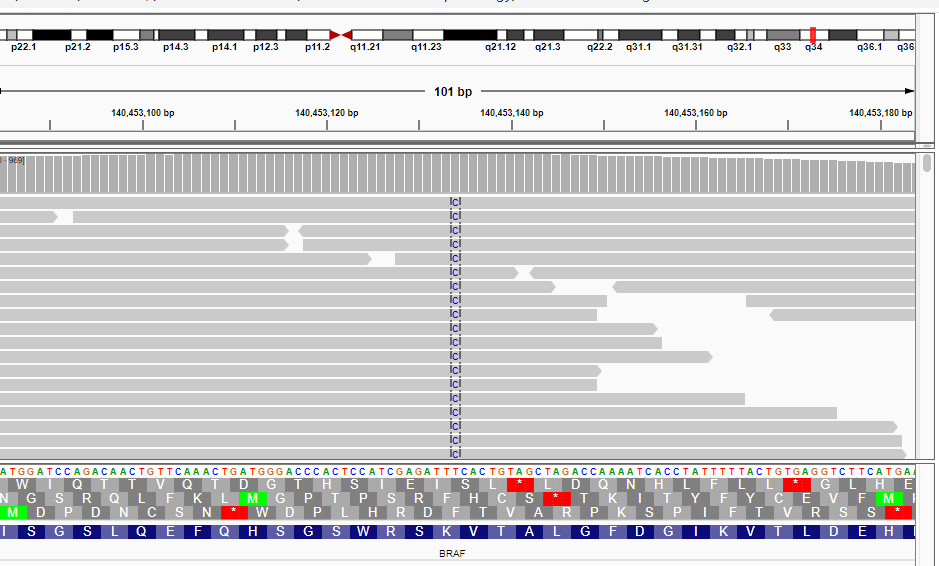


IGV picture of *BRAF* c.1801A>G p.K601E


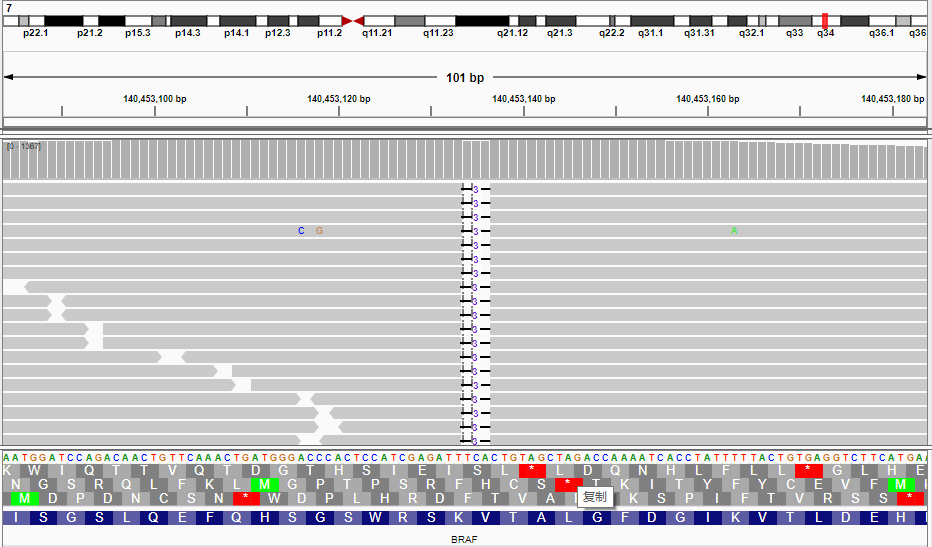


IGV of *BRAF* c. 1799_1801del p. V600_K601delinsE

Supplement: Supplementary file 3 — Additional file 3.IGV pictures illustrating BRAF p.K601E and V600_K601delinsE mutations. [file 13000_2021_1083_MOESM3_ESM.docx]
